# Patients with stress-induced exhaustion disorder and their experiences of physical activity prescription in a group context

**DOI:** 10.1080/16549716.2023.2212950

**Published:** 2023-06-14

**Authors:** Anna Andersdotter Sandström, Anncristine Fjellman-Wiklund, Marlene Sandlund, Therese Eskilsson

**Affiliations:** a Department of Public Health and Clinical Medicine, Sustainable Health and Medicine, Umeå University, Umeå, Sweden; b Department of Community Medicine and Rehabilitation, Physiotherapy, Umeå University, Umeå, Sweden

**Keywords:** Burnout, physical activity, focus groups, goals, grounded theory

## Abstract

**Background:**

Physical activity is a useful means to improve symptoms and memory performance to some extent in individuals with stress-induced exhaustion disorder. Individuals in this group commonly do not need to reach the recommended levels of physical activity. Developing methods to support physical activity as a lasting behaviour is important.

**Objective:**

The aim of the study was to explore the processes involved when using physical activity prescription as part of rehabilitation in a group context for individuals with stress-induced exhaustion disorder.

**Method:**

A total of 27 individuals with stress-induced exhaustion disorder participated in six focus groups. The informants underwent a multimodal intervention including prescription of physical activity. The physical activity prescription had a cognitive behaviour approach and included information about physical activity, home assignments and goal setting. The data was analysed with grounded theory method using constant comparison.

**Results:**

The analysis of the data was developed into the core category ‘trying to integrate physical activity into daily life in a sustainable way’, and three categories: ‘acceptance of being good enough’, ‘learning physical activity by doing’ and ‘advocation for physical activity in rehabilitation’. The informants identified that during the physical activity prescription sessions they learned what physical activity was, what was ‘good enough’ in terms of dose and intensity of physical activity, and how to listen to the body’s signals. These insights, in combination with performing physical activity during home assignments and reflecting with peers, helped them incorporate physical activity in a new and sustainable way. A need for more customised physical activity with the ability to adjust to individual circumstances was requested.

**Conclusion:**

Prescription of physical activity in a group context may be a useful method of managing and adjusting physical activity in a sustainable way for individuals with stress-induced exhaustion disorder. However, identifying people who need more tailored support is important.

## Introduction

Stress-related ill-health dominates in the OECD countries, and mental ill-health accounts for a large proportion of the long-term sick leave and disability. With appropriate actions taken by policy-makers, employers, employment service and healthcare the personal and economic losses [1] may be minimised which makes applicable methods for rehabilitation a global health interest. In Sweden, stress-induced exhaustion disorder (SED) (ICD-10 diagnosis F43.8A) [2] is one of the most common diagnoses [3] and is considered a valid clinical equivalent to burnout [2,4]. The main symptoms are severe exhaustion secondary to at least 6 months of identifiable stress exposure, reduced mental energy and increased need for recovery after mental effort. Patients with SED also suffer from cognitive impairments [4], and usually anxiety, depression [5], sleep disturbances [4] and different kinds of pain [6].

Evidence shows that physical activity is an important measure in the rehabilitation of SED. Individuals who follow recommendations regarding physical activity significantly improve in their symptoms of burnout and depression [7] and in maximal oxygen uptake and episodic memory performance [8]. Recommended physical activity in the prevention and treatment of SED is in line with WHO guidelines for adults: at least 150 min of moderate intensity, at least 75 min of vigorous intensity or a combination of moderate and vigorous intensity physical activity performed at least three times per week [9]. Although physical activity reduces symptoms and improves physical capacity and cognition, individuals with SED have difficulty maintaining physical activity over time [10,11]

Physical activity prescription, which is often used in Swedish healthcare, could increase physical activity [12] and maintain these changes up to 12 months [13]. The main feature is a patient-centred dialogue with focus on behaviour change, individual written prescription in collaboration between the patient and healthcare personnel and is based on current evidence regarding physical activity [13]. The dialogue aims to highlight the patients’ previous experiences of physical activity, current level and identify factors associated to motivation and readiness for behaviour change [14]. Despite this, individual physical activity prescription for people with SED is not adequate support to reach the recommended dose of physical activity or to increase maximal oxygen uptake [8]. Physical activity prescription in a group context has not previously been evaluated.

Because physical activity is a proven method of improving symptoms and cognition in SED, finding methods that can promote more lasting behaviour of regular physical activity is important. Group rehabilitation is shown to support behavioural change [15,16], and the aim of this study was therefore to explore the processes involved when using physical activity prescription as a part of the rehabilitation in a group context for individuals with SED.

## Methods

This study used a qualitative design with an inductive approach based on focus group discussions (FGD) analysed with the grounded theory method (GT) using constant comparison [17,18]. Focus groups were chosen to reach a deeper understanding of the complexity of behaviours and motivation in the process researched, which is possible to achieve through focus group dialogue [19]. The GT method is suitable for a process that evolves over time, resulting in a developed model which we consider is the case in rehabilitation with physical activity prescription for patients with SED [17].

### Study context

The participants took part in a multimodal intervention (MMR) programme including group-based cognitive behavioural therapy (CBT) and individualised workplace intervention to support behaviour changes and return to work. The team involved in the MMR programme consisted of a psychologist or psychotherapist, physician, rehabilitation coordinator and a physiotherapist. The CBT consisted of 23 group sessions and 2 individual meetings to establish and evaluate individual therapy goals for behavioural change. In each CBT group, there were 6–8 participants, and each session lasted for 3 h. In two of the group sessions (sessions 7 and 10), the focus was on physical activity. A physiotherapist led these meetings, and each session lasted for 90 min. In the first session, the physiotherapist provided information about the effects of physical activity and how physical activity can be graded through frequency, duration and intensity to optimise the appropriate level regarding SED [11]. Based on our clinical experience we have noticed that patients with a high degree of anxiety often use physical activity as relief for anxiety. This may reduce symptoms in the short term, but from a longer perspective when the dose of physical activity is too high together with lack of recovery, the symptoms can instead increase. For this reason, graded physical activity is important. The group discussion focused on their thoughts, emotions and behaviours regarding physical activity and current levels of physical activity. Between the two sessions, the group received a homework assignment where the goal was to try different physical activities in everyday life with a variation of intensityfor example, walking, gardening, housework or cycling. The intensity of the activity was recorded either with a pulse monitor or the Borg´s Ratings of Perceived Exertion scale [20]. Most participants chose to use the pulse monitor. In the second session, the homework assignment was followed up and a discussion was held about the physical activity recommendations and motivation regarding physical activity. This resulted in a physical activity prescription where each participant wrote their own individualised goals with the purpose of reaching recommendations for 150 min of moderate-intensity physical activity during a week.

### Participants

Individuals for this study were recruited from a Stress Rehabilitation Clinic. All had a physician-confirmed diagnosis of SED and had undergone an MMR programme. Between May and September 2017, all 49 individuals from eight CBT groups were asked to participate. Twenty-seven individuals from six CBT groups accepted, gave informed consent and were interviewed, 23 women and 4 men (29–63 years of age). Fifteen women and six men (29–65 years of age) declined. Reasons for declining were other commitments, long commute to the location where the interviews were held and that one group engagement in a day was enough (FGDs were held on the same day as their first group reunion after finishing the MMR programme).

### Focus group discussions

The FGDs were held at the rehabilitation clinic 3 months after the MMR programme. The original intervention groups were kept together during the FGDs, and each consisted of 3–6 informants. The FGDs were moderated by the first author and an assistant. The first author asked open-ended questions and led the dialogue; the assistant took notes and made a summary at the end of the FGD. A semi-structured thematic interview guide was created and consisted of the themes of physical activity, physical activity prescription and physical activity that is durable over time.

Each FGD lasted 1–1.5 h, was audiotaped and transcribed verbatim by the first author or a professional transcriber. The transcribed texts were read thoroughly multiple times and compared to the audiotapes by the first author.

### Data collection and analysis

Data was analysed with the GT method using constant comparison [17,18]. We modified the method slightly since time limitations made is impossible to fully analyse each FGD before the next one was conducted. However, the first author listened to every audiotaped FGD, analysed it comprehensively, and if needed made changes to the interview guide to allow the design to grow in accordance with GT. Memos were used throughout the analysis to capture the emergent analysis through initial thoughts and ideas.

Two of the authors (the first and last author) started the analysis by independently reading three of six transcribed FGDs text. The first author analysed the remaining interviews. At first, they coded the transcribed text (line by line), with the data program Open Code [21] and then compered codes and emerging analytical thoughts. Codes with similar content were gathered and grouped into categories and constantly compared with emerging categories. The categories were formed into a core category, and axial coding was implemented when searching for key categories that related to each other. These were compared and a final model was outlined. After the initial coding and at the end of the analysis, the whole research group met to compare and analyse the material. None of the researchers were involved in the informants’ MMR programme.

## Results

**Figure 1.**
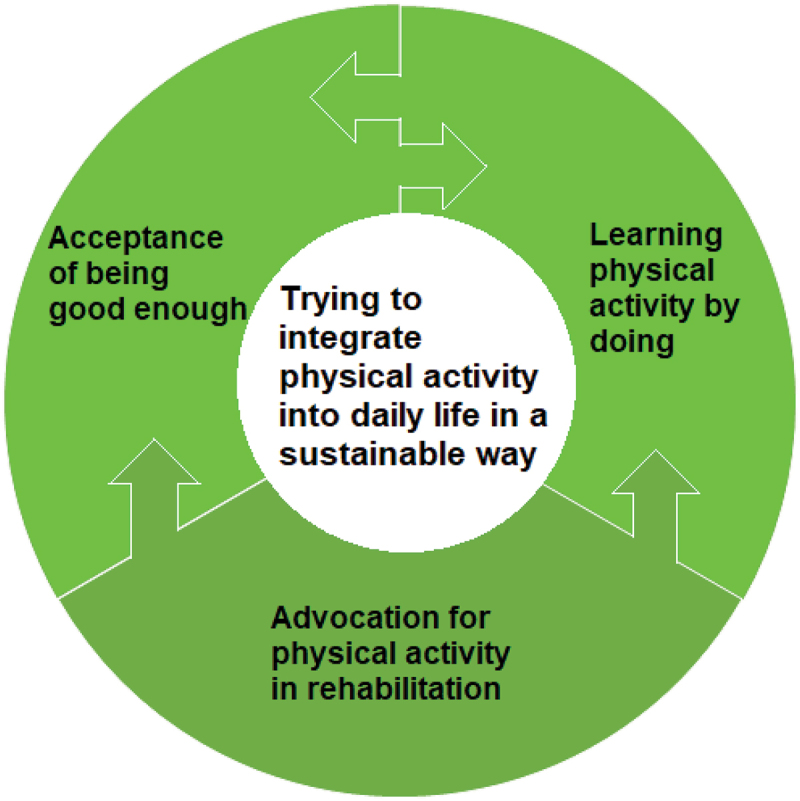
The theoretical model illustrating the core category and the categories.

Analysis of the data resulted in the core category *‘trying to integrate physical activity into daily life in a sustainable way’* and three categories: *‘acceptance of being good enough’*, ‘*learning physical activity by doing’* and *‘advocation for physical activity in rehabilitation’* (Table 1). Our theoretical model (Figure 1) describes the individual’s process during the rehabilitation and the steps towards integrating physical activity into daily life in a sustainable way. Sustainable physical activity means finding a physical activity routine that is possible to maintain over time through a new approach and through adjustment of dose and/or intensity. The informants learned what physical activity was, what was good enough in terms of dose and intensity and how to listen to the body’s signals during the physical activity prescription sessions. This, in combination with performing physical activity with help from the home assignment during which they also discussed their experiences with their peers, helped them incorporate physical activity in a new and more sustainable way over time. The informants adapted the physical activity to their situation and integrated regular activity into everyday life. The intervention was not always enough to help them reach their goals. The need for more customised physical activity was described.

**Table 1.** Patients with stress-induced exhaustion disorder and their experiences of physical activity prescription in a group context. Results by subcategory, category and core category.

Subcategory	Category	Core Category
All types of physical activity counts Listen to the body’s signals	Acceptance of being good enough	Trying to integrate physical activity into daily life in a sustainable way
Try and experience physical activity Reflecting on physical activity with others	Learning physical activity by doing	
Give physical activity importance in the treatment Individualised physical activity	Advocation for physical activity in rehabilitation	

### Acceptance of being good enough

The category *acceptance of being good enough* consists of the subcategories *all types of physical activity counts* and *listen to the body´s signals* and represent how the informants’ knowledge about and attitude towards physical activity changed during the rehabilitation and after the physical activity prescription sessions. The informants asserted that they had new insights which gave them the opportunity to rethink and accept a different level or type of physical activity. Acceptance was identified as the key to be able to make those changes. Since overall energy to spend on different activities was lower, activities needed to be prioritised. To achieve acceptance, the informants had to create a new understanding of what constituted ‘good enough’ of physical activity in relation to their energy. When the informants realised that their own activity levels were ‘good enough’, they had a sense of satisfaction about their physical activity. Their focus changed from performance to something perceived as more sustainable.

The subcategory *all types of physical activity counts* captures the broadened view of the physical activity that the informants acquired during rehabilitation. Before their diagnosis of SED, physical activity was perceived to be higher intensity activities like jogging, running or group training. Outdoor walks were not perceived as physical activity. Previous assumptions about what physical activity entailed were a hindrance to carrying out physical activity. Some were surprised when they discovered, with help from the pulse monitor, that they reached a higher intensity of physical activity than they perceived. When they recognised that certain activities counted as physical activity, it changed their view of their own physical activity levels.

I had to rethink when we talked about physical activity, because I thought I had to do a lot … .when you suffer from stress-induced exhaustion you don’t have the energy. So for me, it was important to hear that walks or biking were counted as physical activity. Because otherwise it feels like I wouldn’t have done anything. – FGD 2

Well then, it might become even more important to lower my goals. It might be five or ten minutes of physical activity at lunch and think that it is okay. – FGD 4

The subcategory *listen to the body’s signals* encompasses the informants’ need to relate to their own body and present symptoms. During the rehabilitation, the informants started to listen to their body’s signals. Examples of signals to listen for were fatigue, muscle tension or heart rate. It helped to be consciously present, both in body and mind, during each activity. They learned how to act according to the body´s signals, that is, to be more physically active or to recover. This made it possible to adjust the activity according to their status of the day and to find a better activity balance.

If I feel stressed out then I can’t go out running, then it won’t be good, it will not fill that function of restoring my energy, it will be the opposite.

What I have become good at during this time is that you have begun listening to your inner voice – how do I feel today? What level should I put it (activity level) on? – FGD 6

It was important to know what to listen to and act upon when listening to the body. To learn bodily signals and to recognise the differences between bodily fatigue, mental fatigue and emotional fatigue was helpful in maintaining physical activity that ‘gave energy’ and did not ‘take energy’. The informants learned that there are different types of fatigue experienced in SED, and that different types of fatigue imply different needs. Sometimes the body needed physical rest, and other times it needed activity.

… .and some days you must take it easier, and other days if there are a lot of thoughts that are spinning the head, more physical activity is needed …. – FGD 6

### Learning physical activity by doing

The category *learning physical activity by doing* is built by the subcategories *try and experience physical activity* and *reflecting on physical activity with others*. This step highlights the way the home assignment was an eye-opener because the informants had to personify their new knowledge and experience. In addition to the physical experience of performing physical activity, the opportunity to reflect on their experiences with others was described as a positive experience since it exposed both similar and different experiences that supported the individual’s own journey.

The subcategory *try and experience physical activity* represents how the home assignment was central in embodying the informants’ new knowledge regarding physical activity. It gave them the opportunity to test physical activity in everyday life based on their own conditions. As a part of the home assignment a pulse monitor was used, so that the informant could validate the intensity of specific activities. The pulse monitor was concrete and showed how the body reacted to different activities. For some, the pulse monitor gave them the courage to try activities and intensities that they previously avoided out of fear of symptoms. To try and experience a physical activity gave the informants confidence to continue physical activity and reduced the amount of avoidance.

I was afraid and felt the heart (rate) rise … when I used the pulse monitor. I realized that it was mostly a feeling and could also note that if I continued and actually listened inwards and persuaded myself that this is nothing dangerous, then I noticed that the pulse got slower and more stable, and I could actually continue the physical activity. – FGD 3

The subcategory *reflecting on physical activity with others* represents the recognition and distinction between the individuals’ experiences during the group discussions. To share experiences with others was articulated as an overall positive experience.

It is really good because you can feel that you’re not alone and you get to hear experiences from the others. Therefore, it’s good that the (physical activity in prescription) is conducted in a group. You might come up with things when someone else is talking, like aha that might also be true.

Another positive thing about the group is that you could be totally honest, you could say anything. Because we are somewhat alike, we are all in the same boat (…) and there is a recognition (…)

You’ve also been inspired when someone report of something that worked out for them. Then just maybe you could try that approach. – FDG 6

It was also communicated that individual differences in the group could be a hindrance. Those with higher levels of physical activity and those with low levels of physical activity were described to be too far apart in experiences, and this made it more difficult to benefit from discussing this together.

…. (there are) so many things in this rehabilitation you can recognize in yourself. It has been nice, to compare yourself with others and see that ‘well, I’m like anyone else’ or ‘everyone also experiences this’. But in the physical activity (experiences), I think that we have been very unlike within this group and that made it harder …. – FGD 3

### Advocation for physical activity in rehabilitation

The category *advocation for physical activity in rehabilitation* includes the subcategories *give physical activity importance in the rehabilitation* and *individualised physical activity*. The category presents the place where the need for individual support was identified. Informants perceived positive effects of taking part in the physical activity prescription group sessions, but there was a desire for concentrating on physical activity, for some it was clear that the focus on each individual disappeared in the group context. This was important to those who already engaged in regular, high physical activity and those who did not reach the recommendations and struggled to do so.

The subcategory *give physical activity importance in the rehabilitation* depicts an opinion that physical activity did not get enough attention in the rehabilitation in relation to other parts of the rehabilitation plan. The informants’ knowledge of physical activity and its effects in stress, anxiety, depression and SED were the reason for their reflections.

Then, also, I would like to have more of this physical activity, because it is so important. – FGD 5

Some informants did not find the physical activity prescription sessions to have any value and described that they had a different focus during the rehabilitation, and it did not include physical activity.

To practice physical activity as a group was suggested as one way to highlight its importance. This was expressed by those who described difficulties in initiating activities, who experienced difficulties in finding new activities, or had limited experience of physical activity.

If physical activity is a way to get healthier, you might need something, a kick in the butt or some kind of group where you could be physically active together or something like that. *–* FGD 2

The subcategory *individualised physical activity* captures differences in how the informants perceived the provided information about physical activity. Some said that the information was just enough for them to be able to absorb. Others voiced that they would benefit from receiving the information on a deeper level.

I think it was a sufficient level so you could understand. At first, I felt that I would have liked even more information, but I understood after the session that it was enough. You can´t absorb more [information] …. – FGD 2

Some of the informants had an individual meeting with a physiotherapist. Individualised physical activity provided an opportunity to address more specific issues related to physical activity. Follow-ups regarding physical activity were expressed as something that would be of use.

I thought that was great since it gave me a reminder and a push, and the physiotherapist had a follow-up [individual meeting with physiotherapist] …. So that made this a lot easier for me to do. – FGD 4

## Discussion

This study fills a knowledge gap regarding how individuals with SED experience physical activity and physical activity prescription in a group context. Our main result demonstrates the individual’s journey towards the goal of the process: to manage physical activity in a sustainable way. Individuals with SED described ‘sustainable’ as a way adopted to current situations and possible to maintain over time. Knowledge of how to make a behaviour change to sustainable physical activity is limited [22,23]. Carlsson et al. [24] defined sustainable physical activity as ‘the possibility to continue the activity for months or even years’. This is in accordance with our results where sustainable physical activity means finding a dose or approach that makes physical activity possible to maintain over time. What our study adds to the definition of sustainability is that it is not just a matter of finding the right dose and frequency. It is also a matter of adjusting the dose in extent and intensity based on the individual’s current condition by increasing or reducing current level. In our study, informants were interviewed 3 months after the end of the MMR programme, and the perception was that they maintained changes until that time. Physical activity prescription in a group context may contribute to maintaining physical activity, which has proven to be a challenge in previous studies [10,11].

Another definition of sustainability regards humanity’s development on Earth. The World Health Organization posted global goals to reach before 2030. One of these goals is to ensure healthy lives and promote well-being for all [9]. Physiotherapists and other healthcare professionals can contribute to this goal through their prevention and health promotion work. For example, a change to more active transportation habits could promote health [25] and decrease greenhouse-gas emissions [26]. In our study, one way to make physical activity sustainable was to learn how to interpret bodily reactions and signals. When the individuals with SED gained those insights, it became possible to adjust physical activity to reach a balanced level. Difficulties in maintaining balanced levels of physical activity can be seen as a result of dysregulation of the autonomic system, meaning elevated sympathetic activity and reduced parasympathetic activity [27]. An autonomic dysregulation is suggested to impair the individual’s capacity for energy restoration [28,29] Reduced mental- and physical energy for individuals with SED might lead to difficulties in finding the right dosage, a balanced dosage, regarding activities in general [11], and physical activity in particular. Earlier research suggests that different activities and intensities place different demands on self-regulatory energy [30]. Balanced energy levels also promote opportunities to engage in regular physical activity [31]. Our clinical experience is that it is important to grade physical activity in case of SED regarding symptoms. For example, an individual with high levels of anxiety might engage in extensive physical activity and needs to lower their dosage of physical activity to maintain balanced energy levels. A study exploring experiences of Qigong with an MMR programme found similar experiences when individuals learned to listen to their bodily signals and acted upon those with a positive outcome [16]. In our study another way to adjust physical activity in a new sustainable way was by using a pulse monitor. This agrees with earlier research that describes technical feedback with pulse monitors as favourable factors and support for individuals with SED [32]. Other studies have found that heart rate monitors [22] or activity tracers [22,33] are useful tools for supporting behavioural changes in physical activity.

A behavioural change support described in this study was the group format. Participants in other rehabilitation group interventions for SED also described a feeling of recognition and that the group members support each other on the path to regain health [16,32]. However, the positive experiences of the group format were not uniform, it could also be a hindrance. The individuals with SED reflected that their differences in experience of physical activity made it more difficult to find parallels with others, and this in turn made it difficult for them to change their own behaviour. A well-known model for behaviour change, the transtheoretical model [34] describes behaviour change as a process involving different stages. Each stage represents different needs in terms of information, support and using one’s own strategies. The model emphasises the importance of meeting the individual’s needs according to which step they are in to allow the process to proceed. Our result demonstrates that individuals with SED had different needs fulfilled during the physical activity prescription process. Identifying where the individual situates themselves in their physical activity process might be key in reaching as many people as possible in a group format; it might also highlight the need for more individualised support for some. To meet the individual’s specific need may require further investigation of the need for more individualised information and customised follow-ups. A comparison of outcome before and after a planned intervention with the use of a control group (RCT) could be a suitable continuation to further investigate this patient group’s need for support and the method physical activity in a group context.

### Strengths and limitations

Strengths of this study include the diversity and depth of data, which suggests that the informants felt safe in expressing their experiences. The study was based on FGDs to reach a deeper understanding of the phenomenon, which is conceivable through group dialogue [19]. Triangulation between researchers was used to increase credibility [18]. Some of the researchers had an insider perspective because of specific expertise and knowledge of rehabilitation for SED; some of the researchers had a more outsider perspective and had expertise on the methods. Study credibility was increased due to prolonged engagement [18] since two of the researchers work at the Stress Rehabilitation Clinic, and thus had a good understanding of the research domain and patient group, but they were not involved in the individuals’ rehabilitation. A peer-debriefing with other professions at the Stress Rehabilitation Clinic and at a conference for physiotherapists was used to increase credibility of the result [18]. A limitation of this study could be that the informants’ prior engagement in the CBT group might affect the data through predetermined roles in the group, and this might influence what topics came up and who spoke the most. The authors' reflection is that informants that are experienced in physical activity might be more prone to confide their thoughts than those with less experience. This limitation was addressed with the participation of an assistant at the FGD, who encouraged each participant to talk and identified questions that were not answered. Another possible limitation of this study is that, even though the informants perceive that their physical activity was sustainable, there is no reliable proof of them doing so. A spreadsheet where the informants documented their physical activity would confirm their statements. The fact that the MMR programme itself might have effects on behaviour change due to the CBT focus must be accounted for when reading this study. However, we believe that results from this study could be applicable in a clinical setting of rehabilitation of individuals with SED and in other stress-related ill health.

## Conclusions

Physical activity prescription in a group context may be a useful method to support individuals with SED in achieving and maintaining physical activity in a sustainable way. The intervention with physical activity on prescription gave individuals new insights regarding their physical activity and new ways to adhere to their individual needs through listening to bodily signals and doing home assignments. The group format was beneficial for most individuals with SED through group discussion and peer recognition. However, it may be important to identify individuals who need more tailored support. When planning MMRs for SED, physical activity should play a central role.

## Paper context

Physical activity is useful to improve symptoms and memory performance in individuals with SED. Individuals in this group commonly do not reach the recommended levels of physical activity. Developing methods to support physical activity as a lasting sustainable behaviour is therefore important. To our knowledge this is the first study that explores the processes involved when using physical activity prescription as a part of rehabilitation in a group context for individuals with SED.
